# Visuo-vestibular integration for self-motion: human cortical area V6 prefers forward and congruent stimuli

**DOI:** 10.1007/s00221-025-07106-8

**Published:** 2025-05-23

**Authors:** Sarah Marchand, Marine Balcou, Philippine Picher, Maxime Rosito, Damien Mateo, Nathalie Vayssiere, Jean-Baptiste Durand, Alexandra Séverac Cauquil

**Affiliations:** Université de Toulouse, CNRS, CerCo, Toulouse, France

**Keywords:** V6, Self-motion, Visuo-vestibular integration, GVS, fMRI

## Abstract

The integration of visual and vestibular input is crucial for self-motion. Information from both sensory systems merges early in the central nervous system. Among the numerous cortical areas involved in processing this information, some (V6 and the ventral intraparietal area –VIP) respond specifically to vestibular anteroposterior information. A series of experiments were carried out to further understand the involvement of these and other areas in self-motion processing when vestibular and visual information are combined with varying congruence and direction parameters. Fifteen subjects underwent an MRI session while receiving visual (optic flow patterns) and galvanic vestibular stimuli, mimicking six conditions: (1) visual forward, (2) visual backward, visual forward with (3) congruent or (4) incongruent vestibular information, visual backward with (5) congruent or (6) incongruent vestibular information. At the voxel-wise level, adding vestibular stimulation to optic flow stimulation activated several bilateral areas located predominantly in the insular cortex. However, the region of interest (ROI) analysis of these areas indicated that none of them exhibits any specificity for the forward/backward direction or for the visuo-vestibular congruency. By extending the ROI approach to other well-known visuo-vestibular areas, we found that the parieto-occipital area V6 is unique in showing not only an increased level of activation for concurrent visual and vestibular stimulation, but also a marked preference when these signals are congruent and specify forward motion. Since area V6 is the only region more active when both visual and vestibular signals specify the most common self-motion direction (i.e. forward self-motion), our results support the view that this area plays a crucial role in visuo-vestibular integration during locomotion. This could be the first step towards the construction of a conscious perception of self-motion, possibly involving other areas.

## Introduction

Monitoring position and motion of our body in space is essential for the control of posture and self-motion. These representations are produced by integrating signals from different modalities, notably of visual, vestibular and muscular proprioceptive origins (Angelaki and Cullen 2008; Britton and Arshad 2019; Pettorossi and Schieppati 2014; Pitzalis et al. 2020). Studies have shown that altered visuo-vestibular integration disturbs self-motion (e.g. Keshavarzi et al. 2023; Schenberg et al. 2023) and can lead to symptoms of nausea, vertigo or dizziness (Bronstein et al. 2020).

Electrophysiological studies in non-human primates have identified several cortical areas in which neurons respond to both visual and vestibular self-motion signals, particularly in the temporal lobe, around both the temporoparietal junction (TPJ) and the midposterior Sylvian fissure. These regions notably include the dorsal part of the medial superior temporal area (MSTd) (Duffy 1998), the ventral intraparietal area (VIP) (Chen et al. 2011a), the visual posterior sylvian area (VPS) (Chen et al. 2011b; Guldin and Grüsser 1998), the frontal eye field area (FEF) (Gu et al. 2016) and parietal region 7a (Avila et al. 2018). In humans, TPJ is well characterized in its role to combine visual and vestibular input into spatial perception and self-motion perception. Transcranial magnetic stimulation (TMS) studies have shown that interfering with TPJ interferes with spatial perception and vestibular processing (Arshad et al. 2014; Kheradmand et al. 2015). In addition, techniques of vestibular stimulation, such as caloric and galvanic vestibular stimulation, have shown TPJ’s function in self-motion perception (Dieterich 2003; Lopez et al. 2008), offering evidence that TPJ is a crucial visuo-vestibular integration center.

Correspondingly in humans, functional imaging studies have revealed that the network of cortical areas responding to both visual and vestibular self-motion signals is extensive (Smith et al. 2017), encompassing the human homologous of the non-human primate region MSTd (hMSTd) (Dukelow et al. 2001), the human homologous of the non-human primate region VIP (hVIP) (Bremmer et al. 2001) and the cingulate sulcus visual area (CSv) (Sluydts et al. 2020). While “VIP” and “MSTd” originally refer to cortical regions identified in non-human primates, functional imaging studies have provided strong evidence for homologous regions in the human brain with similar response properties to multisensory self-motion signals (Bremmer et al. 2001; Dukelow et al. 2001). Therefore, for consistency with previous literature and to align with prior human neuroimaging research (e.g. Bartels et al. 2008; Cardin and Smith 2010), we will refer to the ventral intraparietal region as VIP rather than hVIP and to the dorsal part of the medial superior temporal area as MSTd rather than hMSTd in the remainder of the manuscript.

Overall, both the regions involved (Smith et al. 2018; Cottereau et al. 2017) and their connectivity pattern (De Castro et al. 2021; Sluydts et al. 2020) seem to be highly conserved between these primate species. A notable difference is in area V6, which responds more strongly when optic flow patterns are consistent (rather than inconsistent) with self-motion in humans (Smith et al. 2018) but not in monkeys (Cottereau et al. 2017).

Interestingly, by using galvanic vestibular stimulation (GVS) (Dlugaiczyk et al. 2019) and functional MRI in humans, we recently demonstrated that among the visuo-vestibular regions identified, only two —V6 and VIP areas— respond to GVS when it imitates a motion along the anteroposterior axis, but not when it mimics a lateral motion (Aedo-Jury et al. 2020). GVS has been demonstrated to generate a sense of self-motion by modulating vestibular afferent activity, with the motion being sensed towards the cathode and away from the anode (Fitzpatrick and Day 2004). Electrodes placed on the mastoids in a bipolar configuration, result in a sense of motion dictated by the relative location of the cathode and anode. In the current experiment, we employed GVS in a setup that was arranged to selectively send motion signals in the anteroposterior axis. This approach is based on prior research demonstrating that specific electrode placements can systematically bias perceived motion direction along different spatial axes (Séverac Cauquil et al. 2000). Nevertheless, as stated before, here, the aim is not to generate a conscious perception of self-motion, but only to activate the brain regions involved.

In the present study, we further investigate these observations by combining visual and vestibular signals that can independently specify forward and backward directions along the anteroposterior axis. Our aim is to uncover whether some of the involved regions are more specifically involved in visuo-vestibular integration when visuo-vestibular stimulation mimics ecological conditions of self-motion (*i.e.* when visual and vestibular signals congruently specify forward locomotion).

## Material and methods

### Participants

Subjects were recruited via posters and provided with information about the research, a consent form and a minimum 5-day withdrawal period. Fifteen healthy participants were included in the present study (mean age: 23 years, range: 21–26 years, 10 females, 5 males). All subjects had normal or corrected-to-normal visual acuity. To be recruited, the subjects were required to have (1) no contraindication to MRI, (2) no history of vertigo nor motion sickness, (3) no clinically significant conditions preventing them from staying in the scanner for 1 hour without discomfort, (4) no progressive psychiatric or neurological pathology, (5) a sufficient proficiency in French to understand the consent form and (6) no suspicion of pregnancy. This study was approved by an ethics committee (ID CPP 15-001/2014-A01893-44). All subjects gave their written informed consent before their participation and received 60 euros of monetary compensation.

### Visual stimuli

**Fig. 1:**
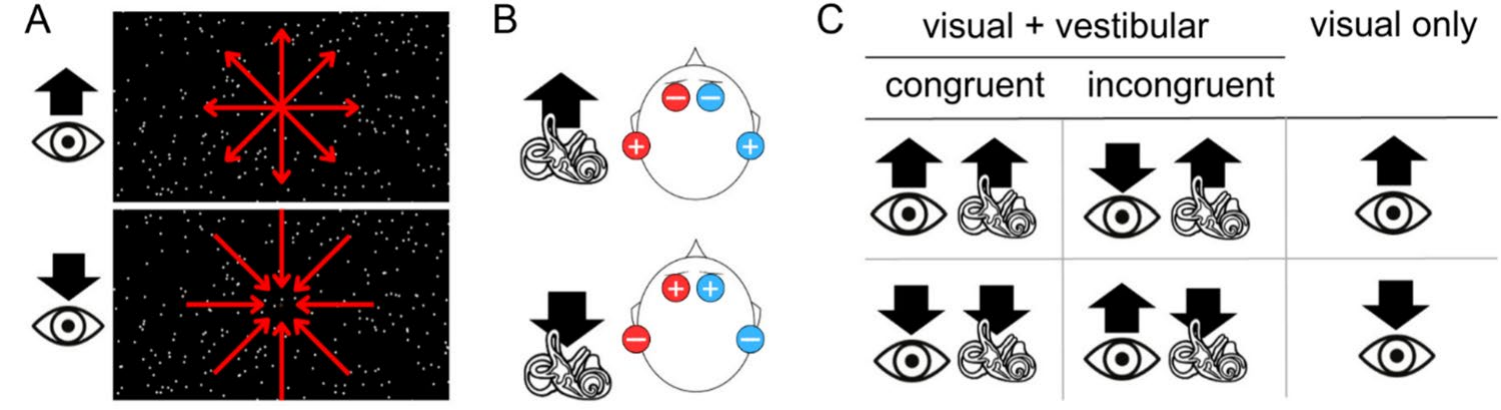
Study paradigm. **a** Visual stimuli. Image captures from the optic flow videos presented to the subjects (superimposed red arrows indicate the expanding or contracting movement of the dots). The large black arrows above the eye logo indicate the direction of the visual self-motion cue (forward for expanding dots and backward for contracting dots). **b** Vestibular stimuli. The two pairs of electrodes, with one anode (+) and one cathode (−) each, are designated by their red and blue colors, respectively. The large black arrow above the vestibule logo indicates the direction of the vestibular self-motion cue (forward or backward). **c** Conditions. The black arrows represent the direction of the specified self-motion direction. The eye represents optic flow stimulation and the vestibule represents GVS stimulation. The experimental design includes six conditions. Two conditions (first column) consisted of multimodal (visual + vestibular) stimulation with congruence in the direction of the self-motion cue (either both forward or both backward). Two conditions (second column) were also multimodal, but with the two modalities being incongruent (one modality specified forward self-motion, while the other signaled backward self-motion. Finally, two conditions (third column) involved only the visual modality, providing a signal of either forward or backward self-motion. During the acquisition, these six conditions were randomly distributed in 60 trials for each run (10 trials per condition) and 8 runs were acquired per subject

The visual stimuli were projected by an overhead projector located outside the scanner room onto a screen at the entrance of the MRI scanner tunnel. A mirror located above the MRI antenna and directed towards the screen at the entrance to the tunnel allowed subjects to see the screen while lying in the scanner. The viewing distance was 82 cm and the diagonal of the monitor was 40 cm long. The stimuli consisted of 2-second of optic flow videos composed of white random dots moving against a black background (Fig. 1a). This short stimulation duration was chosen to avoid motion after-effects and minimize potential postural responses while being sufficient to trigger robust brain activations (Aedo-Jury et al. 2020), notably in area V6 (Cardin and Smith 2011). The videos were displayed at a resolution of 800 x 600 pixels, covering 30 x 23 degrees of visual angle, with a refresh rate of 85 Hz and a motion-coherence level set at 30% (which means largely unambiguous optic flow patterns). To determine the level of motion coherence used in the experiment, we conducted a preliminary study in which subjective vection intensity was assessed in 14 participants for four levels of motion coherence (1; 0.7; 0.5 and 0.3). The results obtained with c = 0.3 were significantly lower than the three other conditions (repeated measures ANOVA, p < 0.001), showing a threshold of motion perception at this level. On the basis of these results, we chose a consistency level of 0.3 for the experiment in order to approach the reliable vection threshold and ensure sufficient sensitivity to reveal multisensory effects. Although the current study does not aim to elicit a conscious perception of self-motion, we considered this threshold in order to place the visual stimulation near the limit at which vection typically emerges, preserving global motion structure. The dots were either expanding to mimic forward self-motion, or contracting to mimic backward self-motion. The focus of flow-field was stable in the center of the screen, and the subjects’ eyes were free to move around, but they had been instructed to look at the middle of the screen, where a fixation cross appeared during the interstimulus interval (ISI).

### Galvanic vestibular stimulation

Before positioning the electrodes, the subject’s skin was prepared with alcohol and the electrodes were covered with conduction gel. As shown in Fig. 1b, the position of the 4 carbon MRI electrodes (Skintact, FSWB00) followed a monopolar binaural setup, with two configurations: 1/to mimic an input of forward motion: the anodes (+) of each of the two pairs of electrodes were placed behind the ear, at the level of the mastoid processes and the cathodes (−) were placed ipsilaterally, on the subject’s forehead, just above the eyebrows 2/to mimic an input of backward motion: anodes (+) of each of the two pairs of electrodes were placed at the level of the forehead and the cathodes (−) were placed ipsilaterally, at the level of the mastoid processes (Séverac Cauquil et al. 1998; Séverac Cauquil et al. 2000). Two stimulators (Digitimer DS5 CE certified for biomedical research N(IEC) 60601) located outside the scanner room, one for each pair of electrodes, delivered the electrical current via MRI compatible cables running through a waveguide. The vestibular stimulation consisted of 2-second of square-wave current of 1 mA intensity between each mastoid process and the subject’s forehead. As with visual stimulation, the stimulation time and intensity were lower than in other studies (*e.g.* Ruehl et al. 2022) to minimize the risks of postural responses, current diffusion and discomfort while being sufficient for triggering robust cortical activations (Aedo-Jury et al. 2020). Subjects reported that neither pain nor discomfort were experienced during the stimulation, which enabled us to avoid the use of potentially allergenic local anesthetics.

### Design

Data were collected using an event-related design. Each run lasted 7.5 minutes. The six defined combinations resulting from the pairing of these two stimulation modalities enabled the creation of two “Congruent” conditions (in which the visual and vestibular organs are simultaneously stimulated by input in the same direction, either forward or backward), two “Incongruent” conditions (in which the visual and vestibular organs are stimulated by input in opposite directions, one forward, the other backward) and two conditions in which only the visual stimulation was delivered (Fig. 1c). In all conditions, the stimulation, whether visual or multimodal, lasted 2 seconds, with an inter-stimulus interval varying randomly between 5 and 7.5 seconds, during which a fixation cross was displayed. The conditions were applied in a pseudo-random order and 8 runs of 60 simulations were performed per subject.

Head position was standardized across all subjects within the MRI scanner. The participant’s head position was controlled according to a strict protocol performed by a trained MRI technician. The participant’s head was stabilized using foam cushions around their head and noise-cancelling headset, and the MRI coil positioned around the subject’s head limited further movement. These measures kept the head as motionless as possible during the acquisition, limiting motion artifacts and guaranteeing consistency in head position between participants.

To ensure participants’ attention throughout the experiment, a motion direction task was included. Participants were asked to maintain their gaze at the center of the screen and to indicate the direction of motion (forward or backward) of the optic flow stimulus. It is important to note that the task was not designed to measure perceptual performance, as the brief duration of the stimulations and the parameters used were not intended to trigger a perceptual response. The purpose of the task was solely to maintain the participants’ attention and ensure they remained engaged with the optic flow stimulus during the experiment.

### Data acquisition

The MRI images were acquired by a clinical 3 T scanner (Philips Achieva), equipped with a 32-channel head coil. At the beginning of each session, a high-resolution T1-3D anatomical image (4 minutes) was acquired (240 slices; repetition time (TR): 7.4 ms; echo time (TE): 3.4 ms; flip angle (FA): 8, percent phase FOV: 100; voxel size 1 x 1 x 1 mm). Functional EPI images for localization of congruent and incongruent visual and vestibular stimuli processing (1 hour) were acquired in the form of 8 runs consisting of 10 blocks of 6 stimuli according to an event-related design, with a TR of 2.5 seconds, a TE of 30 ms, a voxel size of 2 mm, a slice thickness of 3 mm (gap between slices = 0 mm), an FA of 90 degrees, a SENSE factor of 2.8 and an EPI factor of 39. Each run comprised 184 volumes of transversely oriented slices that covered the whole brain.

## Data analysis

### Pre-processing

The functional volumes underwent a conventional pre-processing pipeline, starting with spatial and temporal realignments with the SPM12’s dedicated tools in MATLAB. Volume and slice outliers were detected and repaired using SPM12’s ArtRepair module (Mazaika et al. 2015). The realigned and corrected images were then coregistered to the anatomical image acquired during the same session. The anatomical image then underwent segmentation and normalization in MNI space, and the normalization parameters were finally applied to the functional volumes before spatial smoothing (gaussian kernel, FWHM = 4 mm isotropic). All of the following statistical tests were carried out using the MATLAB interface with the Statistics and Machine Learning toolboxes.

### Whole brain analysis

For each subject, we performed a 1 st level whole-brain analysis using a general linear model (GLM) implemented in MATLAB/SPM12, and then we carried out a 2nd level group analysis for generalization. We mapped the cortical regions whose activation increased when simultaneous vestibular and visual information was provided by contrasting all the “Visual + vestibular” conditions (the 4 conditions in which both modalities were stimulated) with the “Visual only” conditions (the 2 conditions in which only the visual modality was stimulated). We performed statistical inference through cluster-extent based thresholding to correct for multiple comparisons. A voxel-wise threshold of p < 0.001 (uncorrected) was used to identify contiguous clusters and the resulting clusters were then tested against a cluster-extent threshold controlling for cluster family-wise error (FWEc) at p < 0.05 (k = 134; from github.com/CyclotronResearchCentre/SPM_ClusterSizeThreshold/cp_cluster_Pthresh.m). The main activation peaks within these clusters were labeled using the Anatomy3 toolbox with probabilistic cytoarchitectonic maps in the MATLAB/SPM12 interface (Eickhoff et al. 2005), the Brainnetome atlas (Fan et al. 2016) and comparison with similar coordinates in the literature (Frank et al. 2014; Frank et al. 2016; Indovina et al. 2020; Lobel et al. 1998; Wirth et al. 2018) (Table 1). To visualize this functional network on a cortical surface, we used Caret5 software (Van Essen et al. 2001).

**Table 1:** Coordinates of the main local maxima within the insular clusters from the “Visual + vestibular” > “Visual only” contrast, with their peak-level t values and uncorrected p values in the whole-brain second level analysis

#	Label	Anatomy	Left hemisphere				Right hemisphere			
			x	y	z	t and (p) values	x	y	z	t and (p) values
1	AI	Ventral granular insula	− 34	4	− 12	8.64 (p<10^−6^)	38	6	−6	9.03 (p<10^−6^)
2	OP3	Dorsal granular insula	− 38	− 6	4	9.15 (p<10^−6^)	40	− 2	6	6.15 (p<10^−4^)
3	OP8	Frontal opercular cortex	− 32	10	12	10.45 (p<10^−7^)	34	8	14	4.24 (p<10^−3^)
4	PIVC	Dorsal granular insula	− 40	− 10	12	8.16 (p<10^−6^)	36	− 10	16	6.59 (p<10^−5^)
5	OP4	central opercular cortex	−56	2	8	6.67 (p<10^−5^)	54	−10	14	7.22 (p<10^−5^)
6	PIC	postcentral gyrus	− 62	− 16	20	6.63 (p<10^−5^)	60	− 22	22	6.93 (p<10^−5^)
7	PFcm	planum temporale	− 62	− 38	16	5.89 (p<10^−4^)	52	− 32	14	4.82 (p<10^−3^)

**Fig. 2:**
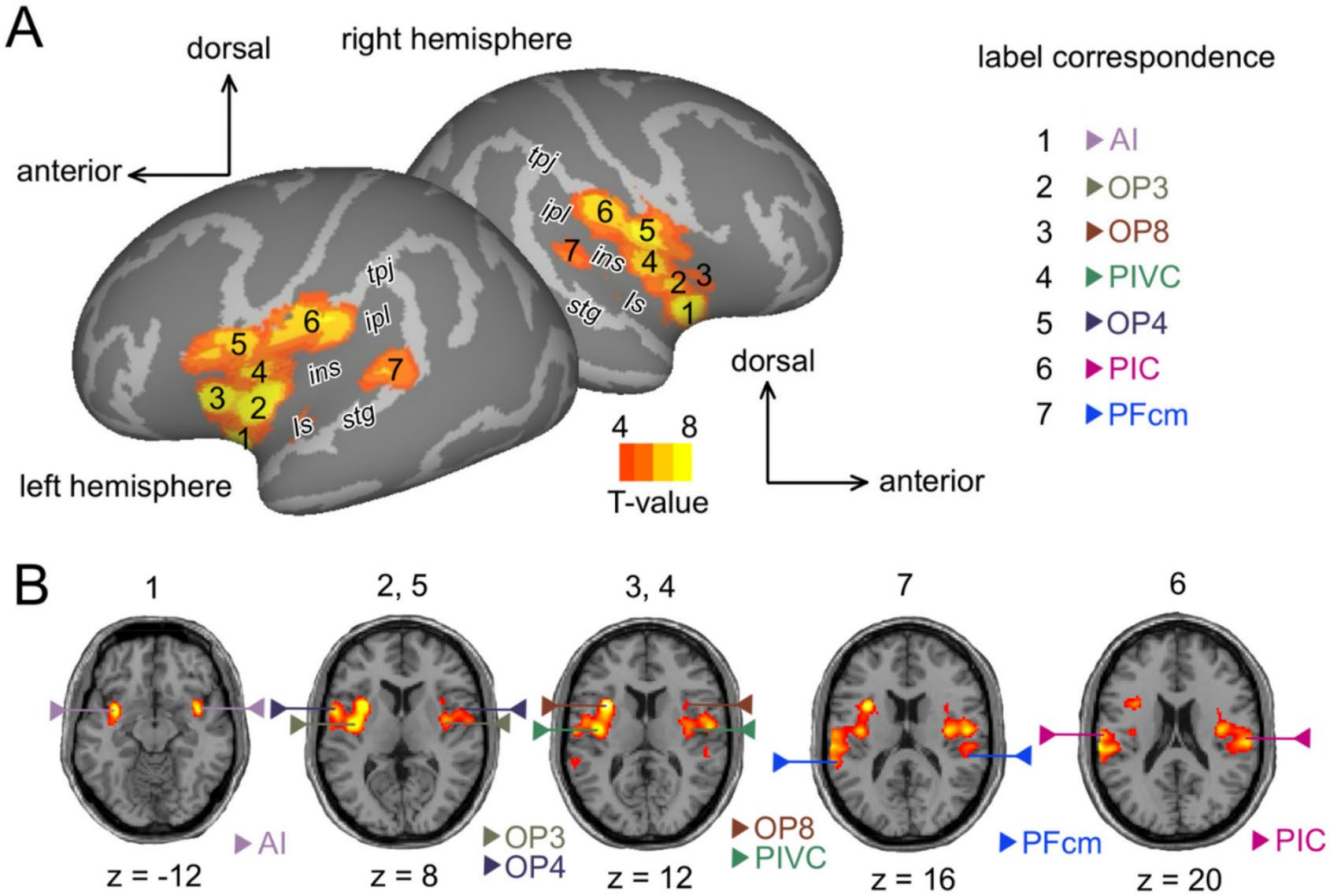
Brain activations for the contrast “Visual + vestibular” > “Visual only”. **a** Statistical parametric map for the contrast “Visual + vestibular” > “Visual only” (second level analysis; n = 15 participants; cluster-extent threshold with p < 0.001 uncorrected and k > 134 voxels) projected on an inflated brain template and on horizontal slices of the SPM12 single subject T1 template. **b** A bilateral cluster centered on the insula exhibited statistical significance in both left (k = 2536 voxels, p_FWEc_ < 10^−15^) and right (k = 1743 voxels, p_FWEc_ < 10^−11^) hemispheres. Within these large clusters, we identified seven regions potentially activated by this general contrast. Coordinates of their local maxima are provided in Table 1, with the associated t values and uncorrected p values. (Anatomical landmarks: *ls* lateral sulcus, *ins* insula, *stg* superior temporal gyrus, *ipl* inferior parietal lobule, *tpj* temporoparietal junction). The number(s) above the slice in (**b**) indicates the number of the region(s) represented. The ends of the lines designate the location of the area and the name of the region is indicated in the corresponding color in the bottom right-hand corner of each slice

The “label” column indicates the name used to designate the corresponding region in this study. The projection on the surface of a template brain as well as the identification of the regions from these coordinates are presented in Fig. 2a and Fig. 2b. *AI* anterior insula, *OP3* operculum 3, *OP8* operculum 8, *PIVC* parieto-insular vestibular cortex, *OP4* operculum 4, *PIC* posterior insular cortex, *PFcm* area supramarginalis magnocellularis columnata (von Economo and Koskinas 1925).

### Region of interest (ROI) analysis

To investigate which of the regions sensitive to the simultaneous solicitation of vestibular and visual modalities (considered here as a positive control) were also sensitive to visuo-vestibular congruence and to the direction of visuo-vestibular input, we used a region of interest (ROI) approach. This analysis was carried out using the coordinates of the regions extracted from the whole brain analysis on the “Visual + vestibular” > “Visual alone” contrast. To avoid statistical circularity, the data set was split in half. ROIs’ coordinates were determined by using the local maxima of the statistical parametric maps obtained with the even-numbered runs for all 15 subjects and their sensitivity profiles were evaluated on the odd-numbered runs only. We generated ROIs in the form of spheres of 5 mm radius (representing 65 voxels) centered on the coordinates of the local statistical maxima using SPM12’s MarsBaR toolbox (Brett et al. 2010). Once the ROIs were obtained, we extracted, for each subject and for each ROI, the mean contrast value for the “Visual + vestibular” > “Visual only” contrast, “Congruent” > “Incongruent” contrast and “Forward” > “Backward” contrast. The “Congruent” > “Incongruent” t-contrast images were obtained by comparing the activations of the congruent conditions (“Visual + vestibular forward” and “Visual + vestibular backward”) with those of the incongruent conditions (“Visual backward + vestibular forward” and “Visual forward + vestibular backward”). The “Forward” > “Backward” t-contrast images were obtained by comparing the activations elicited by forward conditions (“Visual only forward” and “Visual + vestibular forward”) with those of the backward conditions (“Visual only backward” and “Visual + vestibular backward”). Once the mean contrast values had been obtained, we performed t-tests to assess the statistical significance of the contrast of interest for each ROI (one sample t-test, one-tail, hypothesized mean contrast value > 0, significance threshold: p < 0.05 with False Discovery Rate, FDR, correction for the number of ROIs (Benjamini and Hochberg 1995).

In order to determine the effect of our different conditions on regions known to be activated by GVS in the absence of visual stimulation, we conducted a second ROI analysis with the coordinates taken from our previous study (Table 1) (Aedo-Jury et al. 2020) and the methods used to identify these regions are detailed in this previous publication (see Methods section, Aedo-Jury et al. 2020). These coordinates, initially provided in Talairach space, were converted to MNI space using the free online resource MNI <-> Talairach Tool (BioImage Suite, Yale School of Medicine) (Papademetris et al. 2006) (Table 2). We generated spherical ROIs with a radius of 5 mm from these MNI coordinates using MarsBaR (Brett et al. 2010) and we extracted the values from our contrast images in the spheres constituting the ROIs. We used the same contrasts of interest: “Visual + vestibular” > “Visual only”, “Congruent” > “Incongruent” and “Forward” > “Backward” to define the effect of the simultaneous visuo-vestibular information, the congruence of the signals and the direction of the visuo-vestibular input on these vestibular regions (one sample t-test, one-tail, hypothesized mean contrast value > 0, significance threshold: p < 0.05 with FDR correction for the number of ROIs). Since the coordinates were extracted from a previous and independent dataset, eliminating any concerns of data circularity, all the runs were included in this second ROI analysis.

**Table 2:** Coordinates used to generate the regions of interest (ROI) regarding the second ROI analysis on the visuo-vestibular regions

#	Label	Left coordinates			Right coordinates		
		x	y	z	X	y	z
1	V6	− 13	− 86	28	15	− 80	31
2	VIP	− 45	− 43	4	40	− 51	43
3	CSv	− 11	− 27	43	11	− 29	44
4	MT	− 46	− 63	0	43	− 63	0
5	PIC	− 42	− 21	21	43	− 23	18

Coordinates were converted from Talairach (Aedo-Jury et al. 2020) to MNI space (see Methods section). *V6* visual area 6, *VIP* ventral intraparietal area, *CSv* cingulate sulcus visual area, *hMT+* human middle temporal visual area, *PIC* posterior insular cortex.

Note that for all the ROIs considered in these analyses, we first tested for potential differences in activation level for the “Visual + vestibular” > “Visual only” contrast between the left and right hemispheres (paired t-test, p < 0.05 with FDR correction for the number of ROIs). Since none of the ROIs exhibited statistically significant differences, we grouped data from left and right hemispheres to increase the statistical power of our ROI analyses.

## Results

### Visuo-vestibular multimodality network

This study first aims at localizing the cortical regions with increased responses to self-motion signals when vestibular input is concurrent to unambiguous optic flow patterns. To investigate this issue, the brain activations produced when stimulating both the vestibular and visual systems were contrasted with those induced when involving only the visual modality. The results of this contrast are shown on the inflated left and right cortical surfaces (Fig. 2a) and on horizontal sections (Fig. 2b) of template brains (second-level analysis, n = 15, p_FWEc_ < 0.05). The analysis reveals two large and symmetrical clusters centered in the insular cortex and encompassing some portions of the lateral sulcus and temporo-parietal junction (left hemisphere: k = 2536 voxels, p_FWEc_ < 10^−15^; right hemisphere: k = 1743 voxels, p_FWEc_ < 10^−11^). Within these clusters, we identified seven potential bilateral activation sites among which 6 had statistical local maxima below p < 10^−5^ (uncorrected) in at least one hemisphere (p < 10^−4^ for the remaining region). Their coordinates are given in Table 1, together with the corresponding peak-level t and p values. By using the Anatomy3 toolbox (Eickhoff et al. 2005), the Brainnetome atlas (Fan et al. 2016) and a review of the literature regarding the vestibular responsive brain regions, we propose that region 1, which lies in the ventral granular insula, might correspond to the anterior insula (AI) region (Bense et al. 2001; Dieterich et al. 1998; Fasold et al. 2002; Indovina et al. 2020; Wirth et al. 2018). Region 2 is located in the dorsal granular insula and might correspond to area operculum 3 (OP3) (Eickhoff et al. 2005, 2006; Indovina et al. 2020). Region 3, located in the frontal opercular cortex, could match with area operculum 8 (OP8) (Amunts et al. 2010; Eickhoff et al. 2005). A second region located in the dorsal granular insula, region 4, might correspond to previously established coordinates (Frank et al. 2016; Indovina et al. 2020; Wirth et al. 2018) of the parieto-insular vestibular cortex (PIVC) (Grüsser et al. 1990; Guldin and Grüsser 1998; Lobel et al. 1998). It tends to show an anatomical overlap with area operculum 2 (OP2) (Eickhoff et al. 2006; Indovina et al. 2020), often referred to as a major hub in the human vestibular network (Zu Eulenburg et al. 2012; Frank et al. 2014; Frank et al. 2016), activated by vestibular inputs but not by visual motion (Chen et al. 2010; Frank et al. 2016). Region 5, which lies in the central opercular cortex, might correspond to area operculum 4 (OP4) (Eickhoff et al. 2005; Indovina et al. 2020). By the temporo-parietal junction (tpj), located at the lower end of the postcentral gyrus, region 6 coordinates find correspondence with previous studies (Frank et al. 2014; Frank et al. 2016; Wirth et al. 2018) identifying this region as the posterior insular cortex (PIC) (Frank et al. 2014; Indovina et al. 2020). Finally, region 7, located between the superior temporal gyrus (stg) and the inferior parietal lobule (ipl) in the planum temporale gyrus, might correspond to PFcm (area supramarginalis magnocellularis columnata), one of the seven cytoarchitectonically defined “inferior parietal lobule (IPL) areas” (Caspers et al. 2013; Indovina et al. 2020).

While previous research has regularly reported activations in more dorsal parietal regions, such as the angular gyrus and the dorsal supramarginal gyrus, during visual-vestibular stimulation (e.g. Zu Eulenburg et al. 2012; Roberts et al. 2017; Indovina et al. 2020), these did not appear in our “Visual + vestibular” > “Visual only” contrast. There may be several reasons for this. First, our GVS protocol, which was designed to specifically target anteroposterior vestibular cues, may activate a more restricted anterior aspect of the vestibular network, including the insular and opercular regions, as opposed to the dorsal parietal cortices. Second, dorsal parietal cortex may be more active under conditions of perceptual decision-making or active postural control, which were not part of our experimental design. Therefore, while these regions are certainly relevant to visuo-vestibular processing in broader contexts, their lack of activation in the current contrast probably reflects the specific sensory parameters and task requirements of our paradigm.

Note that the opposite contrast (“Visual only” > “Visual + vestibular”) reveals only one bilateral region located close to the central sulcus (MNI coordinates [46 − 10 34] and [− 42 − 10 34]), nearby both the somaesthetic or motor cortices and it corresponds to a region previously described in the literature as bilaterally deactivated when the vestibular saccule is stimulated (Schlindwein et al. 2008).

### Preferences of regions activated by visuo-vestibular stimulation

**Fig. 3:**
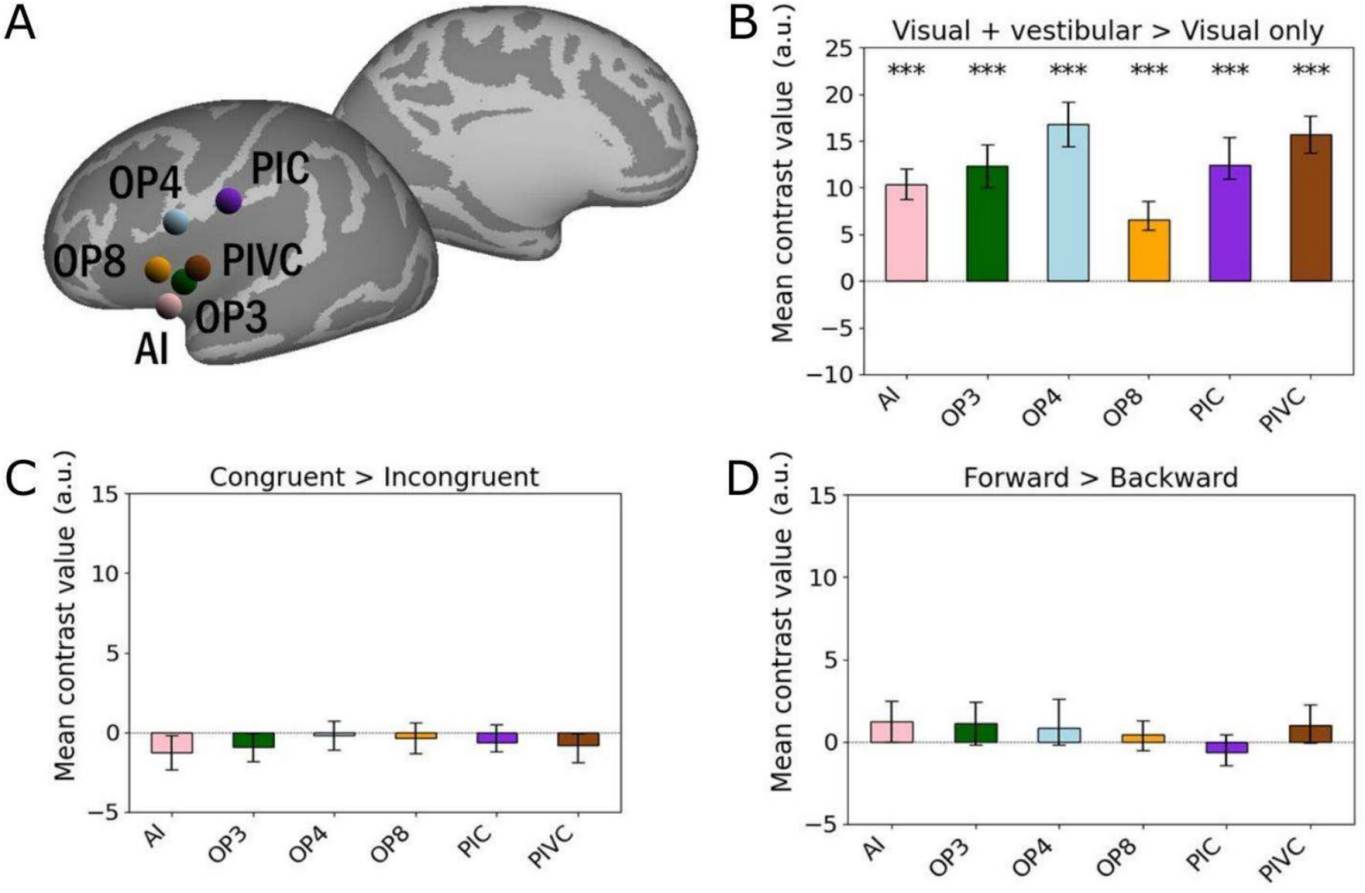
ROI analysis for the regions defined with the “Visual + vestibular” > “Visual only” contrast. Mean contrast value, expressed in arbitrary units (a.u.) within each ROI is the average of that ROI in all hemispheres (n = 30). **a** ROIs’ location on the lateral and medial views of an inflated brain template. Bar plot of mean contrast values in each ROI regarding the “Visual + vestibular” > “Visual only” contrast **b**, the “Congruent” > “Incongruent” contrast **c** and the “Forward” > “Backward” contrast **d**. In all bar plots, error bars indicate the 90% confidence interval (IC90) across hemispheres (n = 30). One-sample one-tail t-tests with the hypothesis that the values are strictly superior to zero were used to assess the significance of each ROI after False Discovery Rate (FDRc) correction of the p values for the number of ROIS (* p_FDRc_ < 0.05, ** p_FDRc_ < 0.01, *** p_FDRc_ < 0.001)

To further investigate whether the above-described regions exhibit a preference for concurrent visuo-vestibular stimulation and could have more specific functions related to the integration of these sensory signals, we performed ROI analyses to assess (1) their change in activation when the stimulation is concurrently visual and vestibular compared to visual only, (2) their preference for congruent visuo-vestibular signals, and (3) their specificity to forward self-motion as specified by these signals. Fig. 3 shows the location of the spherical ROIs on lateral and medial views of the template’s inflated cortical surfaces (Fig. 3a), together with the results of the ROI analyses for the “Visual + vestibular” > “Visual only” contrast (Fig. 3b), the “Congruent” > “Incongruent” contrast (Fig. 3c) and the “Forward” > “Backward” contrast (Fig. 3d). Note that (1) since the activation patterns were visually very similar on both hemispheres (see Fig. 2a and Fig. 2b), left and right ROIs were grouped together to increase statistical power (n = 30 hemispheres), and (2) PFcm (region 7) was excluded from the present analyses because its statistical significance did not hold when using only half of the 15 subjects’ runs. As expected from the voxel-wise analysis, all the regions of interest (AI, OP3, OP4, OP8, PIC, PIVC) show highly significant activations in the “Visual + vestibular” > “Visual only” contrast, with FDR adjusted p values < 10^−5^ in all cases (Fig. 3b). However, none of these regions is significantly activated either for the “Congruent” > “Incongruent” contrast (Fig. 3c), nor for the “Forward” > “Backward” contrast (Fig. 3d).

### Activation preferences of the regions involved in the processing of anteroposterior vestibular input

**Fig. 4:**
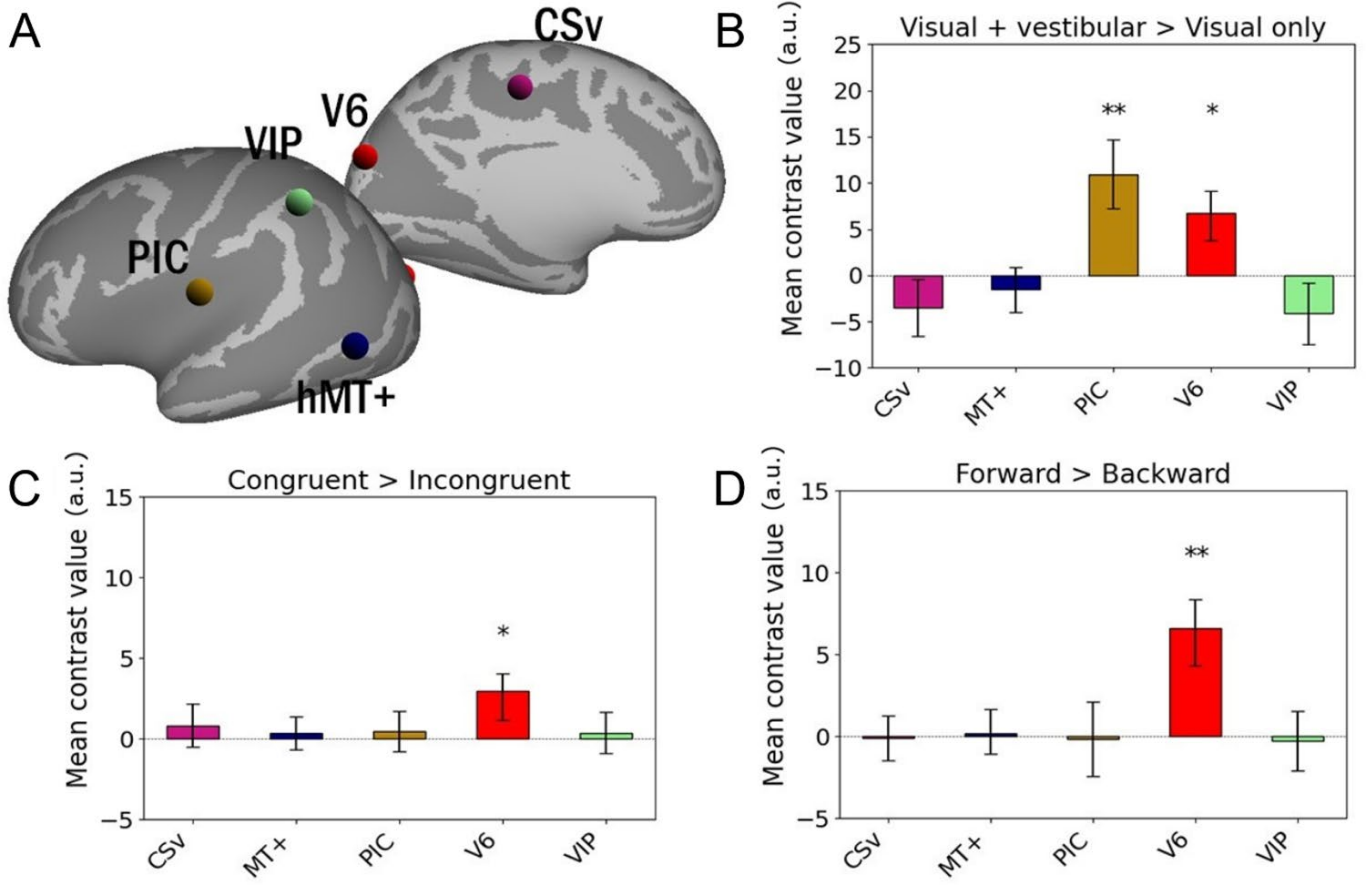
ROI analysis for the regions initially defined in our princeps study (Aedo-Jury et al. 2020) (see Table 2). Mean contrast value, expressed in arbitrary units (a.u.) within each ROI is the average of that ROI in all hemispheres (n = 30). **a** ROIs’ location on an inflated brain template (lateral and medial views). Bar plot of mean contrast values regarding the “Visual + vestibular” > “Visual only” contrast **b**, the “Congruent” > “Incongruent” contrast **c** and the “Forward” > “Backward” contrast **d**. In all bar plots, error bars indicate the IC90 across hemispheres (n = 30). One-sample one-tail t-tests with the hypothesis that the values are strictly superior to zero were used to assess the significance of each ROI after False Discovery Rate (FDRc) correction of the p values for the number of ROIS (* p_FDRc_ < 0.05, ** p_FDRc_ < 0.01, *** p_FDRc_ < 0.001)

Many brain regions known to process both visual and vestibular input (Bruner et al. 2016) were not activated by our main contrast (“Visual + vestibular” > “Visual only”, see Fig. 2a and Fig. 2b). We postulate that with a 30% motion-coherence, the unambiguous and full contrast nature of our optic flow stimuli was sufficient to evoke maximal activations in those regions and that the addition of a simultaneous vestibular input could not further increase the activation level. Nevertheless, we wanted to know whether some of these regions could actually exhibit specific functions related to the visuo-vestibular integration process, by contrast with the vestibular-dominant regions described above. Building on our previous study (Aedo-Jury et al. 2020), in which a visual localizer task was used to identify brain areas responding to optic flow patterns consistent with egocentric movement (Wall and Smith 2008), we constructed ROIs centered in the coordinates given in that previous work for V6, VIP, CSv, the human middle temporal visual area (hMT+) and the posterior insular complex (PIC) (see Table 2 for their MNI coordinates). In the study of Aedo-Jury et al. (2020), V6 and VIP were particularly activated during antero-posterior vestibular stimulation, suggesting a specialization of these regions in motion signal processing in this axis, while CSv, hMT+, and PIC were involved in both antero-posterior and lateral vestibular processing, indicating a more general role in self-motion. Fig. 4 shows the location of the spherical ROIs on the template’s inflated cortical surfaces (lateral and medial view) (Fig. 4a) together with the results of the ROI analyses for the “Visual + vestibular” > “Visual only” contrast (Fig. 4b), the “Congruent” > “Incongruent” contrast (Fig. 4c) and the “Forward” > “Backward” contrast (Fig. 4d), with the same conventions as those used in Figure 3. Regarding the “Visual + vestibular” > “Visual only” contrast, although PIC showed significant activation (p_FDRc_ = 0.004), as expected from the previous ROI analysis, area V6 was also found to exhibit significant activation (p_FDRc_ = 0.010), by contrast with all the other regions considered in this second ROI analysis. In the case of the “Congruent” > “Incongruent” contrast, a single region, V6 again, shows a significant difference (p_FDRc_ = 0.020) between the two conditions, revealing a preference for congruent signals. Finally, in the case of the “Forward” >“ Backward” contrast, the same single region, V6, shows a significant difference (p_FDRc_ = 0.002), indicating that area V6 prefers signals specifying forward self-motion. Therefore, within the 5 regions considered in this second ROI analysis (V6, VIP, CSv, hMT+, PIC) only V6 shows preferential activation when concurrent visual and vestibular signals are congruent and when the direction of the self-motion information is in the forward direction.

## Discussion

This study aimed to more precisely characterize the brain regions involved in integrating visual and vestibular signals, a necessary step towards the perception and control of self-motion. We also addressed their possible specificities regarding signal congruence and direction of self-motion cue. Our results revealed a consistent bilateral network that is particularly engaged when the stimulation is both visual and vestibular. This network is composed of cortical areas (PIVC, PIC, OP3, OP4, PFcm and AI) previously proposed to be parts of the multimodal vestibular cortex (Indovina et al. 2020) and a lesser-known region, OP8, whose role in multisensory integration or self-motion processing had not been reported so far. These results reinforce and complete the current knowledge regarding the mapping of the network involved in the multimodal processing of sensory information linked to the perception and/or control of self-motion (Chen et al. 2011b; Ruehl et al. 2022). Our subsequent ROI analysis indicate that none of the above-mentioned areas show any differential activity either between congruent and incongruent visuo-vestibular information, or between forward and backward self-motion cues (Fig. 3). Nevertheless, these results do not rule out the possibility that underlying neurons can actually be sensitive to these factors but simply that their overall activity does not reflect any global preference for congruence of forward self-motion. We extended our study by repeating these ROI analyses on other regions known to be involved in the processing of both visual and vestibular information. The regions concerned by this second ROI analysis are V6, VIP, CSv, MT+ and PIC (Aedo-Jury et al. 2020) (see Table 2). When the stimulation is visuo-vestibular and not solely visual (Fig. 4b), two of these five regions, V6 and PIC, show significantly greater activation, indicating that they are the most influenced by the concurrent presence of visual and vestibular input. Concerning the discrimination of congruent and incongruent conditions (Fig. 4c), only one region, V6 again, shows significantly more activation in the “Congruent” conditions compared to the “Incongruent” conditions. In the voxel-wise analysis, area V6 did not exhibit statistically significant activation for the “Visual + vestibular” > “Visual only” contrast (cf. Fig. 2), in apparent contradiction with the results reported in this ROI analysis. This is likely explained by the fact that many conditions of visuo-vestibular stimulation included in this general contrast are suboptimal for area V6 (backward and incongruent self-motion signals) so that this area reach the statistical threshold only when it benefits from the higher statistical power offered by grouping individual voxels within ROIs and by combining these ROIs across the left and right hemispheres (grouping voxels lowers the inherent noise level and mixing both hemispheres doubles the sample). Therefore, V6 seems to be specialized in detecting the congruence of visuo-vestibular signals, a function previously unknown to this region. To our knowledge, the only previous study examining the cortical response to congruence or incongruence of visual and vestibular signals in humans was conducted by Roberts et al. (2017), which found preferential activation of the PIVC region in the case of signal incongruence. However, the aforementioned study focused on movement input towards the left or the right, not in the anteroposterior direction, and used caloric vestibular stimulation (Lopez et al. 2012) rather than GVS, suggesting that our new results are not contradictory but complementary. Last but not least, regarding the discrimination between forward and backward self-motion, the V6 area is again the only region to exhibit a significantly greater activation when the visuo-vestibular stimulation mimics forward self-motion (Fig. 4d). Therefore, this region appears to be even more specialized than we initially thought (Aedo-Jury et al. 2020), showing a marked preference not only for the anteroposterior axis, but for the forward direction.

### Magnetic vestibular stimulation

Magnetic vestibular stimulation (MVS) is an inherent effect of MRI, as the strong static magnetic field causes vestibular sensations such as nystagmus or spatial bias (Roberts et al. 2011; Mian et al. 2013). Although this phenomenon is well documented, very recent studies have nuanced its impact, highlighting that behavioral support for MVS-induced vestibular responses remains limited (Bouisset, Nissi, Laakso et al. 2024). Since MVS is constant throughout the acquisition, its impact cannot be expected to provide systematic contrasts across conditions. Our within-subject design ensures that any potential effect of MVS is equally present across all conditions, reducing its impact on the specific effects of vestibular and visual stimulation. Nevertheless, future studies could explore the interaction between MVS and external vestibular stimulation, by including additional control conditions or by testing participants outside the MRI environment for comparison.

### Visuo-vestibular signals integration

The processing of visual stimulation consistent with self-motion is known to engage, in humans, areas such as V6 (Pitzalis et al. 2010), VIP (Bartels et al. 2008), CSv (Wall and Smith 2008), PIC (Sunaert et al. 1999) and MT+ (Morrone et al. 2000). These areas are found in complementary studies that reinforce the strength of their involvement in the perception of self-motion (Cardin and Smith 2010; Wada et al. 2016; Serra et al. 2019) and have also been shown to be activated by vestibular galvanic stimulation (Aedo-Jury et al. 2020). GVS montage selection also significantly influences cortical activation patterns observed in fMRI studies of self-motion processing. The anteroposterior (AP) montage used here was shown to elicit strong activations in area V6, in eyes-closed subjects, whereas the standard lateral left-right montage is associated with an absence of response within the area (Aedo-Jury et al. 2020). This selective activation shows that V6 is particularly sensitive to vestibular anteroposterior axis signals, in line with the established involvement of this area in the processing of optic flow and self-motion during forward walking (Pitzalis et al. 2010). CSv, an area that was found to encode a broad range of self-motion-related information, is activated by both AP and lateral GVS stimulation (Aedo-Jury et al. 2020). This asymmetry likely mirrors the various functional specializations of these regions: V6 is mainly specialized in visual-vestibular integration of forward movement, whereas CSv appears more broadly committed to vestibular encoding of self-motion regardless of the direction of stimulation. These previous findings served as the basis for defining the regions of interest (ROIs) in the present study, which investigates cortical responses under visual-only and combined visual-vestibular conditions. However, a direct comparison between the present results and those reported in Aedo-Jury et al. (2020) is not feasible, given that the earlier study did not include any visual stimulation. These findings emphasize the importance of considering stimulation protocols when interpreting vestibular activations within the human brain. Nevertheless, the strong V6 responses observed with AP GVS in the absence of visual input provide a rationale for exploring visual-vestibular integration specifically along the anteroposterior axis in the present protocol. These findings collectively emphasize the importance of considering stimulation montages and sensory context when interpreting vestibular activations in the human brain.

However, it is worth noting that when we analyze the “Visual + vestibular” > “Visual alone” contrast, out of the mentioned regions, PIC, PIVC and V6 are the only regions that appear to respond more strongly when the stimulation is visuo-vestibular and not only visual (see Fig. 3 and Fig. 4). As only signals of motion in the anteroposterior axis were probed, it cannot be ruled out that some regions within the visuo-vestibular cortical network are not sensitive to self-motion cues in the anteroposterior axis, but are instead dedicated to other axes. Hence, PIC, PIVC and V6 are three sites of visuo-vestibular integration sensitive to the presence of concurrent vestibular information, even when the visual signal is clearly salient. As for the other regions identified as visuo-vestibular but not observed on our contrast, we suggest that they are less sensitive to the presence of a simultaneous vestibular input and a salient visual signal is sufficient to saturate their activation. In other multisensory stimuli, such as visual-auditory stimuli, it has been shown that compensation between the two modalities was most apparent when one of the stimuli was ambiguous (Wada et al. 2003). Consequently, it is likely that under conditions where visual stimulation compatible with self-motion would be less salient, vestibular compensation would be more readily identifiable. Recent psychometric studies have established that a clear vestibular signal can resolve ambiguous visual motion (Alais et al. 2021), and that the visuo-vestibular integration could follow a weighted-averaging rule that depends upon the relative reliability of each modality (Koppen et al. 2019). Further studies are needed to confirm this hypothesis.

Interestingly, despite previous studies showing activation of the most dorsal aspects of the inferior parietal lobule, i.e. the angular gyrus and dorsal parts of the supramarginal gyrus (e.g. zu Eulenburg et al. 2012; Roberts et al. 2017; Indovina et al. 2020), we did not observe substantial activation of these regions in our visuo-vestibular contrast. There are several possible reasons for this absence. Firstly, the GVS protocol used here was configured to preferentially stimulate the anteroposterior axis, and may therefore have more selectively engaged insular and opercular regions as opposed to the more associative parietal cortices. Secondly, dorsal parietal involvement in visuo-vestibular integration may be more important when perception or motor responses are involved, which was not the case in the present protocol.

To our knowledge, similar approaches have not yet been implemented in the context of MRI acquisitions to identify the cortical correlates of this phenomenon. Thus, generating optic flow stimuli with a lower percentage of motion-coherence might provide better visualization of cortical activation in response to vestibular modality intervention. We believe that it would be useful in future protocols to include one or more conditions (depending on the number of axes and directions selected) involving only the vestibular modality. This additional condition would complement the proposed contrasts and would enable the use of tools as the multisensory enhancement index, “which compares the bimodal response to the largest unimodal response” and the additivity index, “which compares the bimodal response to the sum of the unimodal responses” (DeAngelis and Angelaki 2012; Stein and Stanford 2008). It should be noted that the present study investigates the integration of simultaneous visual and vestibular signals at the cortical level, deliberately without reporting any perceptual aspect of the subject. Our aim here was to decipher the mechanisms involved in integration following simultaneous stimulation of the visual and vestibular organs, and not in response to the subject’s own perception of self-motion. The cortical response to the conscious perception of simultaneous visual and vestibular stimuli could be studied in a similar way to ours, using longer and/or more intense stimuli, but deserves further research with an adapted protocol (Ruehl et al. 2022).

One limitation of the current investigation is the absence of a “vestibular-only” condition, where GVS would have been delivered in the dark to induce vestibular activations independent of visual stimulation. This condition, although performed in our previous study (Aedo-Jury et al. 2020), would have provided valuable information regarding the specific vestibular contributions. Time limitations due to the length of acquisition within the MRI scanner did not allow us to include this condition within the current experimental protocol. The cortical responses of isolated vestibular stimulation compared to simultaneous visual and vestibular signals remains uncertain, and any subsequent work could involve such a condition so as to enable the definition of vestibular response more precisely, particularly in regions such as the insular cortex that are known to exhibit vestibular-related activations. This approach would provide a deeper understanding of the independent and collaborative functions of visual and vestibular information in the processing of self-motion signals.

### V6 involvement in self-motion signals integration

Our results reveal a great and unique specificity of area V6 in the processing of self-motion information in the anteroposterior axis, particularly in the context of congruent visuo-vestibular signals and in the forward direction. While the evolution of the human brain and visuospatial integration is also a subject of study in the field of anthropology (Bruner et al. 2016), it is not surprising to imagine that cortical regions such as V6 are particularly specialized in the processing of information related to self-motion in the most ecological situation for humans (i.e., forward movement with visuo-vestibular stimulation). Through visual testing paradigms of self-motion perception, V6 was quickly recognized as an important region in the integration of sensory information useful for locomotion and also visually guided eye, hand and foot movements (see: Fig. 2 in Bellagamba et al. 2022;Sulpizio et al. 2023), responding well to optic flow patterns while also showing a sensitivity to stereoscopic depth gradients (Cardin and Smith 2011; Cardin et al. 2012). Subsequent studies further documented its involvement in the multisensory integration of signals linked to self-motion, with vestibular (Aedo-Jury et al. 2020; Cardin and Smith 2010) and proprioceptive sensitivity (Schindler et al. 2018), as well as in the differentiation of self from object motion (Sulpizio et al. 2024). V6 is undoubtedly a key region for the multisensory integration of information necessary for locomotion.

Recent comparative studies of the macaque and human brain demonstrated intriguing similarities in the V6 areas of both species, particularly with regards to functional features and anatomical composition. Macaque and human V6 regions both have large visual receptive fields, high directional selectivity of motion, and complete representation of the visual field, including the far periphery (Pitzalis et al. 2013). These shared features emphasize the primary role of V6 in processing optic flow, particularly that produced by self-motion. In macaques, V6 has been uniformly implicated in the analysis of self-motion by optic flow, a fact further supported by functional studies in animals (Galletti et al. 1999). These findings support the notion that the V6 areas of macaques and humans are involved in the same neural process for movement-related visual information, providing a basis for direct comparison between the two.

Within the present study, we make an important contribution to the understanding of its role by highlighting, for the first time, a specialization for ecologically-meaningful self-motion signals; that is, signals that are congruent across modalities and specify the most common —forward— direction of self-motion.

## Conclusion

In this study, we document a consistent bilateral cortical network with increased activity when vestibular self-motion signals coincide with visual ones. Importantly, we also demonstrate that area V6 plays a pivotal and unique role in visuo-vestibular integration within the context of ecologically relevant forward self-motion. These findings provide new insights into our understanding of cortical self-motion processing and multisensory integration and may help in comprehending the pathologies associated with deficient visuo-vestibular integration.

## Data Availability

No datasets were generated or analysed during the current study.
